# Prevalence of burnout among German radiologists: a call to action

**DOI:** 10.1007/s00330-024-10627-5

**Published:** 2024-02-12

**Authors:** Moritz B. Bastian, Laureen Fröhlich, Joel Wessendorf, Michael Scheschenja, Alexander M. König, Jarmila Jedelska, Andreas H. Mahnken

**Affiliations:** Department of Diagnostic and Interventional Radiology, University Hospital Marburg, Philipps University of Marburg, Baldingerstrasse 1, 35043 Marburg, Germany

**Keywords:** Professional burnout, Radiology, Prevention, Occupational health

## Abstract

**Objectives:**

In the presence of escalating global concerns regarding physician burnout, this study aims to analyze the prevalence and associated factors of burnout among radiologists in Germany.

**Methods:**

A comprehensive online survey, inclusive of 73 targeted questions including a German-modified version of the Maslach Burnout Inventory, was distributed among all members of the German Radiological Society and the German Interventional Radiological Society between May and August 2023. The survey encompassed aspects of employment, workload, well-being, and coping mechanisms. Data from 172 completed surveys were analyzed, with correlations explored via crosstabs and the Pearson-chi-square test.

**Results:**

In total, 76.7% of participating radiologists were identified to be burnt out. The prevalence was significantly associated with increased workload, reduced sleep quality, suboptimal working conditions, reduced job satisfaction, and the negative interplay between work, family life, and health. Median work satisfaction was described as “satisfied” while median workload was assessed as “frequently overwhelming of work.” A total of 41.9% of respondents noted facing daily time pressure. Radiologists’ concerns about work interfering with private family life were voiced by approximately 70%, and 73.3% highlighted the perceived negative effects on their health.

**Conclusion:**

The pronounced prevalence of burnout among German radiologists demonstrates an urgent, unmet need for comprehensive interventions and systemic changes. Our findings act as a catalyst for initiating targeted, multifaceted strategies and dialogs, essential for fostering a resilient and effective healthcare ecosystem. Further large-scale systematic studies should follow to analyze the findings in broad.

**Clinical relevance statement:**

Consistent with other countries, there is a high prevalence of burnout among radiologists in Germany. A call for further investigation is recommended to help mitigate adverse outcomes associated with physician burnout.

**Key Points:**

*• The prevalence of burnout has yet not been evaluated for German radiologists.*

*• German radiologists have a high prevalence of burnout.*

*• Steps must be implemented to engage this problem to prevent worsening.*

**Graphical abstract:**

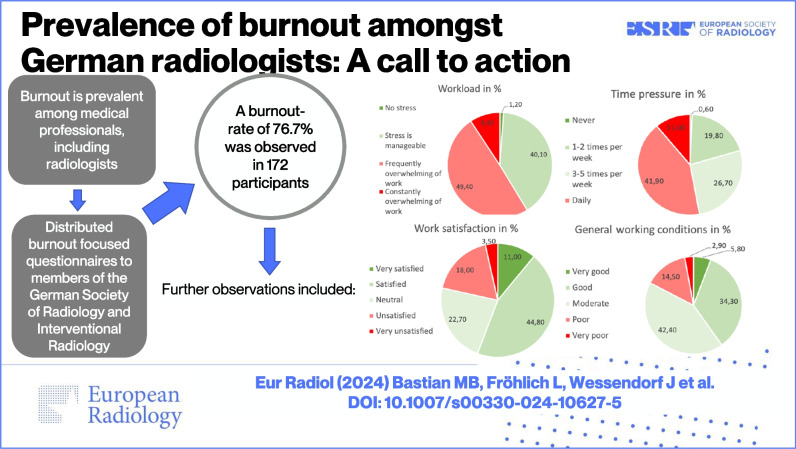

**Supplementary information:**

The online version contains supplementary material available at 10.1007/s00330-024-10627-5.

## Introduction

In the ever-evolving landscape of modern healthcare, the well-being of medical professionals has taken center stage due to its profound implications for both practitioners and the patients they serve. One of the pressing challenges in this context is work satisfaction and burnout, a multifaceted phenomenon stemming from chronic workplace stress that is unmanaged [1]. Characterized by emotional exhaustion, depersonalization, and a reduced sense of personal accomplishment [2], burnout not only endangers the mental and physical health of physicians but also poses a significant threat to the quality of patient care and overall effectiveness of healthcare systems [3]. Beyond its impact within the workplace, burnout has been associated with a cascade of problems that extend to the personal lives of practitioners. These issues encompass a wide range of challenges including deteriorating physical health, disturbances in sleep patterns, and strained interpersonal relationships, all of which culminate in a diminished quality of life.

Studies have indicated that burnout is disproportionately high among physicians, and radiologists are no exception [4–6]. Recent research conducted in the UK highlighted a concerning prevalence of burnout among interventional radiologists of 71.9% (*n* = 244) [7]. Moreover, previous studies originating from the USA and Canada already displayed the global nature of the issue, revealing alarming burnout rates among both diagnostic and interventional radiologists [8–13]. These localized findings are undermined by a comprehensive meta-analysis that encompassed a diverse spectrum of medical disciplines, demonstrating a pervasive burnout challenge among physicians at large [14].

Given the critical associations of physician burnout for both medical practitioners and patient care, this study sets out to investigate the prevalence of burnout and associated risk factors among radiologists in Germany. The scope of this study encompasses all radiological disciplines, including diagnostic, interventional, neuroradiology, and pediatric radiology. A current study evaluated the work expectations, their fulfillment, and exhaustion among radiologists in Germany [15], but not the psychological syndrome of burnout. However, our study seeks to evaluate the prevalence of radiologist burnout and the intricate interplay between the burnout and potential risk factors within the radiological field of the German healthcare landscape. By doing so, it aspires to contribute to a deeper understanding of the factors potentially contributing to burnout among radiologists and to lay the groundwork for targeted interventions that bolster the well-being of these healthcare professionals.

## Materials and methods

### Study design

The questionnaire consisted of 73 questions aiming at the following categories: demographic data, employment status, specialty, on-call and out-of-hours on-call duties, workload, well-being (health, sleep quality, private life, etc.), and adaption to/coping with workload. Question types included yes/no, multiple choice, and Likert-scale grading questions, respectively. The 22 last questions were a German-modified version of the Maslach Burnout Inventory [16], having in mind that the Maslach Burnout Inventory is regarded as the gold standard for burnout assessment. The burnout questions are divided into three dimensions: emotional exhaustion (nine questions), depersonalization (five questions), and personal accomplishment (eight questions). A score of ≥ 10 was considered “burnout.” The Maslach Burnout Inventory is a validated questionnaire, while the other 51 questions, unvalidated and created for the study, are provided in the supplementary file. The study was in accordance with the ethical standards of the institutional research committee and with the 1964 Helsinki Declaration and its later amendments or comparable ethical standards. The study received approval in form of a waiver from the local ethical committee.

### Data collection

The study period was from May 2023 to August 2023. Data was collected prospectively. The online questionnaire was sent to members of the German Radiological Society (DRG) via their monthly newsletter, as well as to members of the German Interventional Radiological Society (DeGIR). DRG and DeGIR together have around 12,000 members.

Study data were collected and managed using REDCap (Research Electronic Data Capture) tools hosted at our university. Data remains private on our university servers, which are compliant with the German General Data Protection Regulation (GDPR) and prevent the sharing of data with third parties. REDCap is a secure, web-based software platform designed to support data capture for research studies, providing (1) an intuitive interface for validated data capture; (2) audit trails for tracking data manipulation and export procedures; (3) automated export procedures for seamless data downloads to common statistical packages; and (4) procedures for data integration and interoperability with external sources [17, 18].

### Statistical analysis

Statistical analysis was performed with Statistical Package for Social Sciences (SPSS, Version 29, IBM Corp.); hereby, a *p*-value of ≤ 0.05 was considered to be statistically significant. Descriptive statistics was done to evaluate demographic data and frequency rates of answers. Correlation analysis of burnout with other categories was done via crosstabs and the Pearson-chi-square test to explore significant correlation. *t*-test was conducted to analyze potential differences among the subspecialties. M.B.B. is the statistical guarantor of the study.

## Results

### Demographics

A total of 218 people started the survey, whereas 172 people completed the survey. Only completed surveys were used for analysis. Roughly 2% of all members from DRG and DeGIR took part in the survey. Demographic data can be seen in Table 1. The employment status varied from full-time (69.2%), part-time (28.5%), honorary basis (0.6%), to self-employed (1.7%). Full-time participants worked median 49–59 h per week, and part-time participants worked median 40–48 h per week.

**Table 1:** Displayed are the demographic data in percent (%) of 172 study participants in the categories sex, age, professional status, professional field, and workplace

Demographic data	Percent
Sex:	
-Male	51.2%
-Female	48.35%
-Non-binary	0.45%
Age:	
- < 30 years	16.3%
-31–40 years	40.7%
-41–50 years	22.1%
-51–60 years	12.2%
- > 60 years	8.7%
Professional status:	
-Resident	30.2%
-Consultant	25%
-Senior consultant	35.5%
-Head of department	8.1%
-Others	1.2%
Professional field:	
-Diagnostic radiology	83.7%
-Neuroradiology	9.3%
-Interventional radiology	26.2%
-Pediatric radiology	9.3%
Workplace:	
-University hospital	34.9%
-Local hospital	29.7%
-Private hospital	11.0%
-Church-related hospital	9.9%
-Outpatient clinic	12.8%
-Other institution	1.8%

### Work situation

**Fig. 1:**
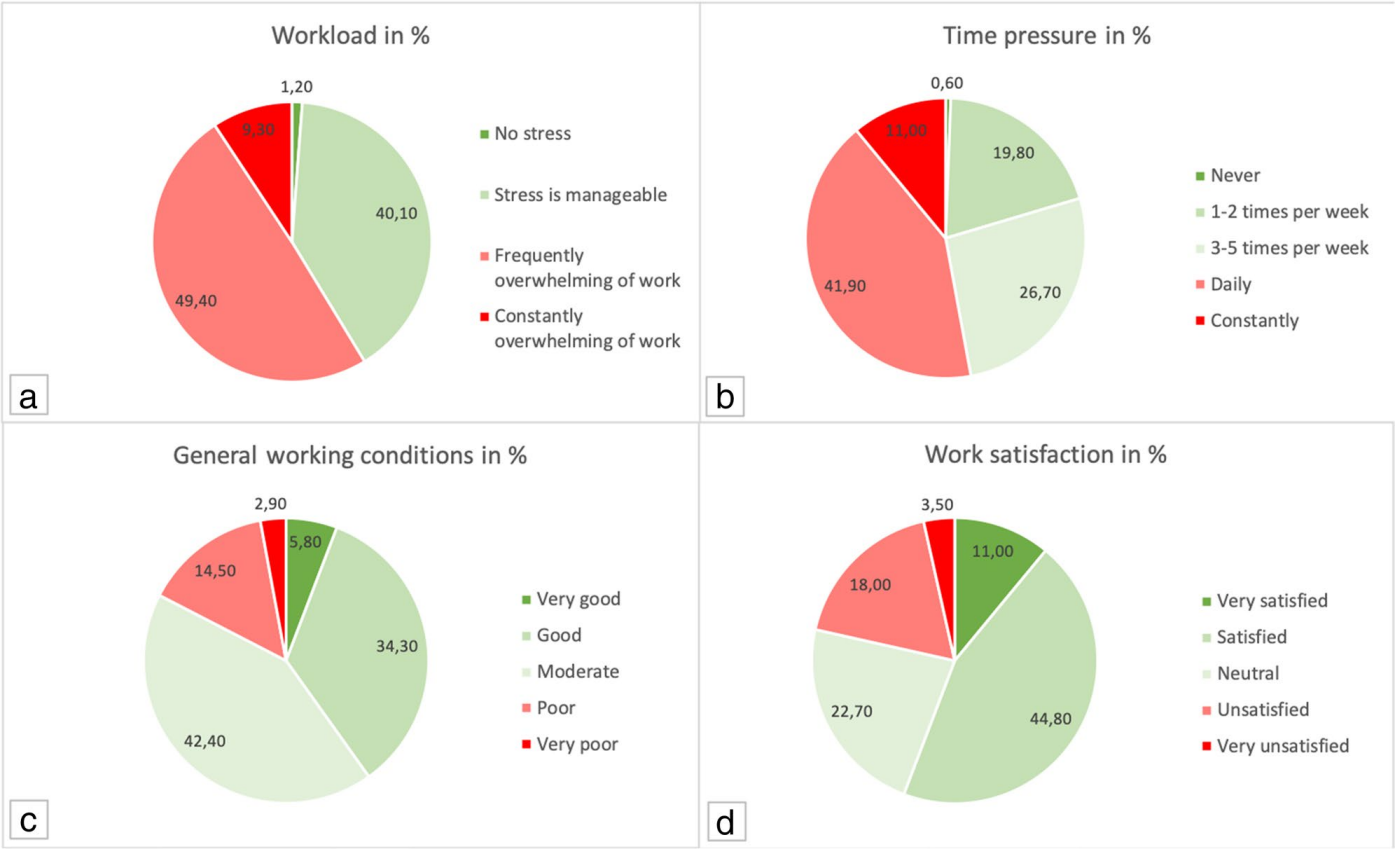
Displayed are the questionnaire answers of the 172 study participants in percent (%) for the following questions: **a** “How do you estimate your workload?” **b** “How often per week do you experience time pressure?” **c** “How do you assess your current working conditions?” **d** “How satisfied are you with your work?”

Residents stated to have on-call duty median 3–4 times a month. Other employees than residents stated they had median 1–5 on-call duties per month. Five to nine extra-hours per week are frequently done by all participants in the median, while 23.4% stated there is no declaration of overtime. Of those who declare overtime, they either get paid (34.8%), compensatory time-off (32.3%), or no compensation (32.9%). Participants prefer in 72.3% of cases to get compensatory time-off instead of being paid. The relief by non-physician staff was stated in 52.9% of participants. Teamwork with non-physician staff was stated as “good” in the median and “good to very good” in the mean. Workload was median stated as “frequently overwhelming of work.” Daily time pressure during work was stated by 41.9% and 84.3% sacrifice their break at work (25.6% daily, 39.5% weekly, 19.2% monthly). General working conditions were seen as moderate in the median and moderate to good as mean (Fig. 1).

### Well-being

Work satisfaction was “satisfied” in the median and “neutral to satisfied” in the mean. Roughly 70% of participants stated that their work would interfere with their private family life and 73.3% stated that their work would influence their health in a negative way.

Mean and median sleep quality was “moderate to good” and “moderate,” respectively. Roughly 60% stated that they should take better care of their health. Regarding the feeling of being burnt out, it was median rarely, but frequent in 31.8% of participants. Only 16.9% reported never to have depressive symptoms. Drug intake to cope with psychic stress was affirmed by 12.8% of radiologists. Around 17.4% of radiologists stated that they already had to undergo treatment due to psychological stress/burnout, while 8.1% have the diagnosis of burnout. Regarding the consideration to leave the physician profession, 59.6% have either had this thought or currently have it. Separately, 22.8% of all surveyed physicians are actively contemplating a change of profession, while 5.3% are considering a shift in their specialty.

### Burnout

A German-modified version of the Maslach Burnout Inventory (with its three dimensions: emotional exhaustion, depersonalization, and personal accomplishment) was used for evaluation of radiologist burnout. Emotional exhaustion had a mean score of 4.94 and a median score of 5. Depersonalization had a mean score of 3.52 and a median score of 4. Personal accomplishment had a mean score of 5.2 and a median score of 5. The total burnout score had a mean value of 13.74 with a median value of 14. The mean burnout scores for the radiological subspecialties were 13.38 for diagnostic radiology, 14.53 for interventional radiology, 12.44 for neuroradiology, and 16.38 for pediatric radiology. There was no significant difference between the subspecialties (Table 2.). In total, 76.7% of participants had a score greater than or equal to 10. Crosstabs and the Pearson-chi-square test were used for correlation analysis. A significant correlation could be seen between burnout with the following categories: increased workload (*r* = 0.320, *p* < 0.001), reduced sleep quality (*r* = 0.280, *p* < 0.001), suboptimal working conditions (*r* = 0.364, *p* < 0.001), reduced job satisfaction (*r* = 0.443, *p* < 0.001), increased time pressure (*r* = 0.240, *p* = 0.001), negative interplay between work/family/health (*r* = 0.261 and *r* = 0.333, *p* < 0.001), burnout diagnosis and treatment (*r* = 0.239, *p* = 0.002), self-perception of burnout (*r* = 0.443, *p* < 0.001), symptoms of depression (*r* = 0.388, *p* < 0.001), the intention to leave medical practice and/or change of career (*r* = 0.286, *p* < 0.001).

**Table 2:** German-modified Maslach Burnout Inventory scores in total and in the radiological subspecialties (diagnostic, interventional, neuroradiology, and pediatrics) divided in quantity, mean, median, and participants with scores > 10 in %. *p*-value displays the results of *t*-test analysis for significant differences between subspecialties

	**Total**	Diagnostic	Interventional	Neuroradiology	Pediatric
Quantity	**172**	144	45	16	16
Mean	**13.74**	13.38	14.53	12.44	16.38
Median	**14**	14	16	13	16
Score > 10	**76.7%**	74.3%	84.4%	81.13%	100%
*p*-value		0.152	0.196	0.395	0.090

## Discussion

Our study unveiled the alarming prevalence of burnout among radiologists in Germany, echoing an increasingly widespread concern in the international radiological community. Drawing from a diverse respondent base, it was found that 76.7% of participants had burnout, as evidenced by scores of ≥ 10 on the German-modified version of the Maslach Burnout Inventory. This prevalence was significantly correlated with a variety of factors, including workload, sleep quality, working conditions, job satisfaction, and negative impacts on family life and health. These findings illustrate the multifaceted nature of burnout and its profound implications on the professional and personal lives of radiologists.

Previous results from burnout studies on radiologist from the UK, the USA, and Canada [7, 8, 13, 19] mirror the burnout prevalence findings of our study, reinforcing the global nature of this issue. Our study further shows the intricate correlations between burnout and various aspects of professional and personal well-being, echoing similar sentiments of strain and exhaustion reported internationally. Comparing the results to national data on the prevalence diagnosed in Germany (which showed a 4.2% prevalence [20]), a roughly two-fold higher prevalence of diagnosed burnout was observed in our study. While the explicit mention of diagnosed burnout is uncommon in literature, it is important not to leave this information unaddressed. When compared to other medical specialties, radiologists have the fourth highest burnout prevalence (46%) as shown by a Medscape survey among physicians in the USA [21]. Unfortunately, a national analysis on the prevalence of burnout highlighting the different medical specialties has yet not been conducted in Germany.

The adverse effects of burnout on healthcare professionals not only impact their personal well-being but also have consequences on patient care. Central to the manifestations of burnout are emotional exhaustion and depersonalization. These symptoms, in turn, may lead to diminished empathy in clinical encounters. Consequently, patients may perceive a decline in the quality of care, feeling less understood and less valued in their interactions with burnt out healthcare providers. Additionally, burnout can be conceptualized as a vicious cycle within the medical community: as burnout is associated with increased illness and absenteeism among physicians [22–24], the workload and pressure on their still-working colleagues will intensify. This escalation in workload subsequently possibly elevates the risk of burnout, which possibly leads to a cycle of negative feedback, where the quality of care decreases, and work morale suffers.

In terms of job satisfaction, it is noteworthy that while the working conditions may be described as only moderate, many physicians expressed satisfaction with their professional roles. This could be attributed to the fact that the dimension of emotional exhaustion is more likely to contribute to their burnout. This might make their current status appear challenging or undesirable from an external perspective. Therefore, their satisfaction, thus, might not necessarily be a testament to optimal conditions but rather a reflection of their personal accomplishment by having faith that their career is making a difference in patient care.

However, the study is not without limitations. The reliance on self-reported data could potentially be subject to bias, as participants might either underreport or overemphasize their levels of well-being and satisfaction due to social desirability or recall bias. While the Burnout Maslach Inventory is widely regarded as the gold standard for evaluating burnout, it is important to note that professional assessment should always complement it. This is essential because the test may exhibit a negative trend in some cases, and a comprehensive evaluation by a healthcare professional could provide a more accurate and holistic understanding of an individual’s burnout status. Additionally, the use of an online questionnaire and the specific outreach methods may have introduced a selection bias, as those who are more inclined to participate might have specific experiences or views on the issue of burnout. Furthermore, the study was prone to response bias, as those with burnout may have been more likely to respond, and additionally, radiologist may have been so burnt out that they did not have the energy to participate in the study. Lastly, the response rate was not at large compared to the possible participants; nevertheless, this study is the first of its kind in Germany and will potentially encourage further research on this important topic in the German radiological community.

In conclusion, the prevalence of burnout among radiologists in Germany emphasizes a pressing need to address this issue for the advancement of clinical practice and patient care. This study gives a first impression on burnout among German radiologists and motivates to conduct further systematic research on this topic. Interventions tailored to alleviate excessive workload, improve working conditions, and bolster overall well-being have begun to become a crucial aspect [25, 26], however still have areas of improvement as displayed by the study. The complex correlations between professional challenges and personal well-being emphasize a need for a holistic approach in addressing burnout. The insights gained from this study not only contribute to the growing body of knowledge on this issue but should also further sensitize the radiological medical community, policy makers, and healthcare institutions. The aim should be to create collaborative paths in alleviating the impacts of burnout, fostering a more resilient, effective, and passionate healthcare landscape for radiologists.

### Supplementary information

Below is the link to the electronic supplementary material.

Supplementary file1 (PDF 133 KB)

## Abbreviations

DRG: German Radiological Society
DeGIR: German Interventional Radiological Society
REDCap: Research Electronic Data Capture

## Declarations

### Guarantor

The scientific guarantor of this publication is Prof. Dr. Andreas H. Mahnken.

### Statistics and biometry

No complex statistical methods were necessary for this paper.

### Study subjects or cohorts overlap

No study subject or cohort overlap has been reported.

### Methodology

• prospective

• observational

• performed at one institution
