# Supplementary material for: Prevalence of burnout among German radiologists: a call to action
Source: Eur Radiol. 2024 Feb 12;34(9):5588–94. doi: 10.1007/s00330-024-10627-5 (PMC11364704; doi:10.1007/s00330-024-10627-5)
Supplement: Supplementary file 1 — Supplementary file1 (PDF 133 KB) [file 330_2024_10627_MOESM1_ESM.pdf]

Translated questionnaire exclusive of the German modified version of the Maslach Burnout Inventory

Personal Data:
- What gender do you identify as? Female, Male, Non-binary, Other

- How old are you? < 30, 31-40, 41-50, 51-60, >60

- In which country do you work? Germany, Switzerland, Austria, Other

- What is your professional position? Resident Physician, Specialist Physician, Senior Physician, Chief Physician, Other

Occupational status:
- What is your area of employment? University Hospital, Municipal Hospital, Church related Hospital, Private Hospital, Other Inpatient Facility, Outpatient Facility, Other

- How many beds does your employer have? < 200, 200-400, 401-800, >800, none

- How are you employed? Full-time, Part-time, Freelance, Self-employed

- For part-time: What is your employment in hours per week (including all shifts and overtime)? 1-9h, 10-19h, 20-29h, 30-39h, >39h

- For part-time: What is your actual employment in hours per week (including all shifts and overtime)? 5-9h, 20-29h, 30-39h, 40-48h, 49-59h, 60-79h, >80h

- For part-time: What are your preferred working hours per week (including all shifts and overtime)? 5-19h, 20-29h, 30-39h, 40-48h, 49-59h, >60h

- For full-time: What are your actual working hours per week (including all shifts and overtime)? 30-39h, 40-48h, 49-59h, >60h

- For full-time: What is your preferred employment in hours per week? >30h, 40-48h, 49-59h, >60h

Duties and Overtime:
- How many on-call duties do you have per month on average? None, 1-2, 3-4, 5-6, >7

- Resident Physician: How many on-call duties do you have per month? None, 1-2, 3-4, 5-6, >7

- How many standby duties do you have per month? None, 1-5, 6-10, 11-15, >16

- How many overtime hours do you work per week? 1-4h, 5-9h, 10-19h, >20h, None

- Is there a time recording for your work? Yes, Electronic, Yes, Handwritten, No

- How is your overtime compensation handled? Paid, compensatory time-off, Neither

- How do you prefer your overtime compensation? Paid, Time off in lieu, I don't want compensation

Experience in Radiology:
- What is your primary area of employment? (Multiple answers possible) Diagnostic Radiology, Neuroradiology, Interventional Radiology, Pediatric Radiology

- How many years have you worked in diagnostic radiology? 1-3, 3-5, 5-7, 7-10, >10, 0

- How many years have you worked in neuroradiology? 1-3, 3-5, 5-7, 7-10, >10, 0

- How many years have you worked in interventional radiology? 1-3, 3-5, 5-7, 7-10, >10, 0

- How many years have you worked in pediatric radiology? 1-3, 3-5, 5-7, 7-10, >10, 0

Workload:
- How much time do you spend daily on administrative tasks? 1 hour, 2 hours, 3 hours, ≥4 hours, 0 hours

- Are you relieved by non-medical staff? Yes, No

- How do you assess your collaboration with nursing staff? Very good, Good, Average, Poor, Very poor

- How do you estimate your workload? No stress, Stress is manageable, Frequently overwhelming of work, Constantly overwhelming of work

- Have you ever filed an overload report? Yes, No

- Have you noticed a change after filing the overload report? It got better, It stayed the same, It got worse

- How often per week do you experience time pressure? Never, 1-2 times per week, 3-5 times per week, Daily, Constantly

- Do you forego your break during working hours? Yes, daily; Yes, more often per week; Yes, occasionally per month; No

Well-being:
- Does your work consume you to the extent that it affects your private and family life? Yes, No

- Do you feel that the scheduling of your working hours affects your health (e.g., sleep disturbances, frequent fatigue)? Yes, No

- How do you rate the quality of your sleep? Very good, Good, Moderate, Poor, Very poor

- Have you been so psychologically burdened by your work that you had to seek medical/psychotherapeutic treatment (e.g., due to burnout)? Yes, No

- Do you pay enough attention to your own health? I pay attention to my health, I should pay more attention, I am very negligent with my health

- How do you assess your current working conditions? Very good, Good, Moderate, Poor, Very poor

- How often do you experience the feeling of burnout? Never, Very rarely, Rarely, Regularly, Frequently, Very frequently

- How often do you experience symptoms of depression (e.g., sadness, lack of motivation)? Never, Very rarely, Rarely, Regularly, Frequently, Very frequently

- Have you ever taken medication due to (psychosocial) stress? Yes, No

- How satisfied are you with your work? Very satisfied, Satisfied, Neutral, Unsatisfied, Very unsatisfied

- Have you ever considered giving up/abandoning your medical profession? Yes, No

- Have you already received a diagnosis of burnout syndrome? Yes, No

Adaptation to Workload:

- How often do you extend your working hours? Never, Very rarely, Rarely, Regularly, Frequently, Very frequently

- How often do you increase your work pace? Never, Very rarely, Rarely, Regularly, Frequently, Very frequently

- How often do you reduce the quality of your work? Never, Very rarely, Rarely, Regularly, Frequently, Very frequently

- What are the reasons for extending your working hours, increasing your work pace, or reducing the quality of your work? (Multiple answers possible) Staff shortage; Increased workload; (Time) pressure Administrative burden/documentation requirements; Other

- Are you considering a career change? Yes, to another specialty; Yes, to another profession; No
